# Supplementary material for: Boosting the Capacitive Performance of Supercapacitors by Hybridizing N, P-Codoped Carbon Polycrystalline with Mn3O4-Based Flexible Electrodes
Source: Nanomaterials (Basel). 2023 Jul 12;13(14):2060. doi: 10.3390/nano13142060 (PMC10383068; doi:10.3390/nano13142060)
Supplement: Supplementary file 1 [file nanomaterials-13-02060-s001.zip › nanomaterials-2477817-supplementary.pdf]

Supplementary Materials

Boosting the Capacitive Performance of Supercapacitors by Hybridizing N, P-Codoped Carbon Polycrystalline with Mn_3O_4-Based Flexible Electrodes

Yu-Min Kang and Wein-Duo Yang *

Department of Chemical and Materials Engineering, National Kaohsiung University of Science and Technology, Sanmin District, Kaohsiung City 807, Taiwan; f110146142@nkust.edu.tw

* Correspondence: ywd@nkust.edu.tw; Tel.: +886-73-81-4526 (ext. 15116)

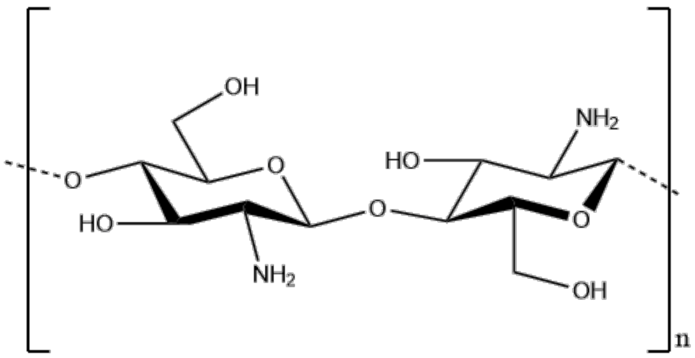

Figure S1. The chemical structure of chitosan.

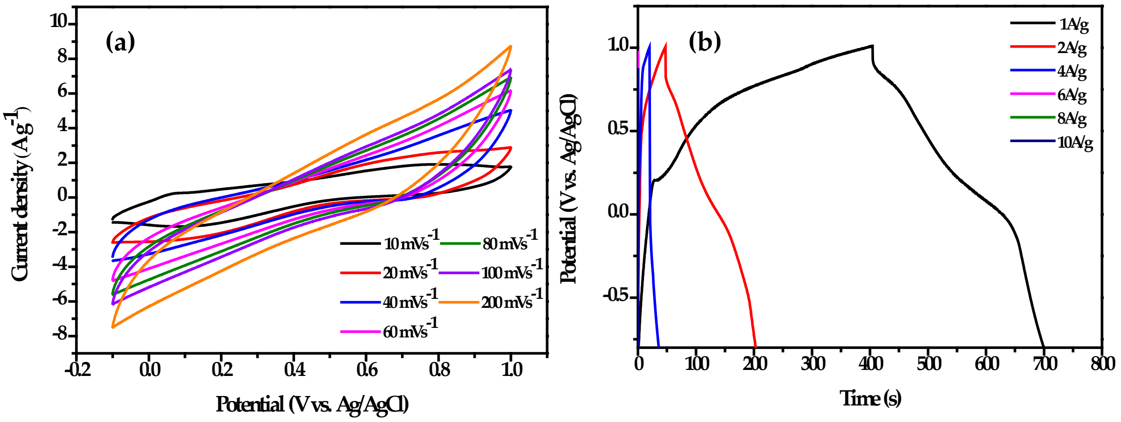

Figure S2. The Electrochemical proerties of the as-fabricated Mn_3O_4@NPC electrode by adding 15 ml H_3PO_4 as P-dopant source. (a) CV examination and (b) GCD test (C_m = 172 F/g at 1 A/g).

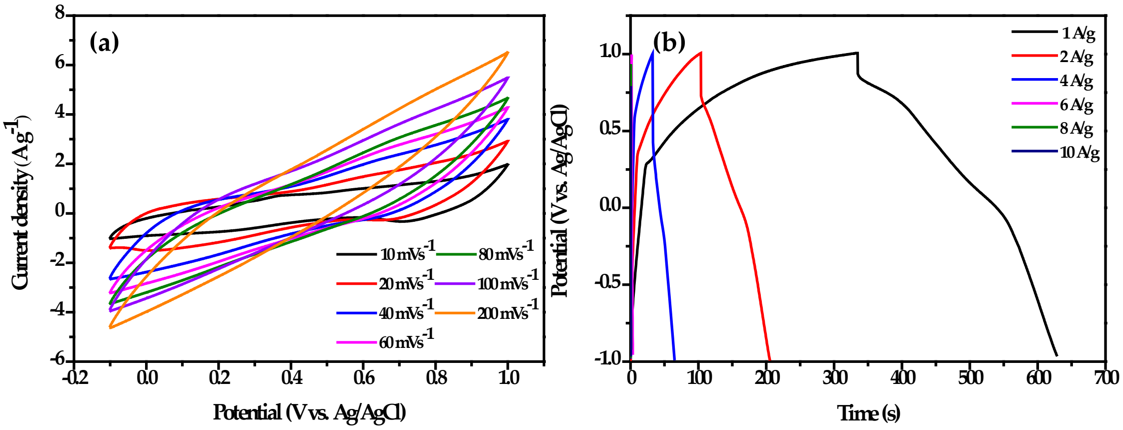

Figure S3. The Electrochemical proerties of the as-fabricated Mn_3O_4@NPC electrode by adding 20 ml H_3PO_4 as P-dopant source. (a) CV examination and (b) GCD test (C_m = 155 F/g at 1 A/g).

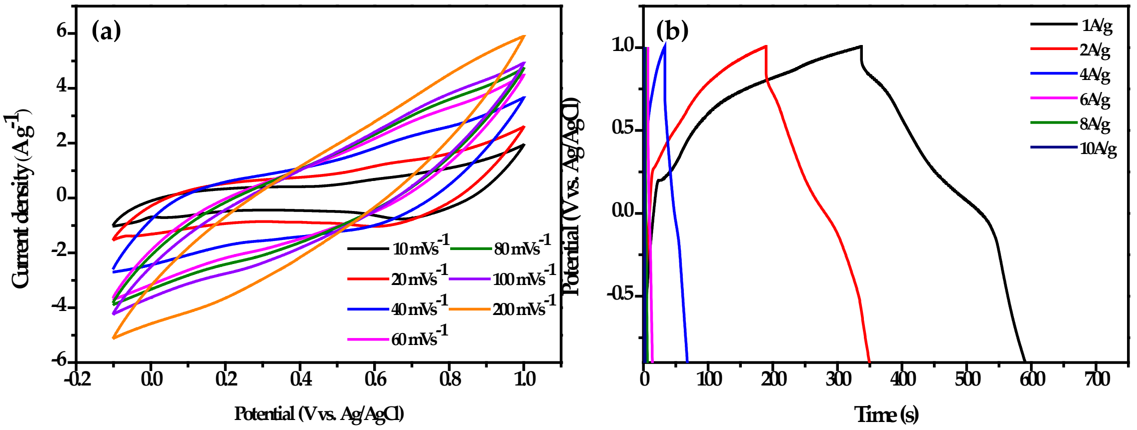

Figure S4. The Electrochemical proerties of the as-fabricated Mn_3O_4@NPC electrode by adding 25 ml H_3PO_4 as P-dopant source. (a) CV examination and (b) GCD test (C_m = 141 F/g at 1 A/g).
